# Is age still a barrier in ventricular tachycardia ablation? A study of safety, efficacy and clinical outcomes in the elderly

**DOI:** 10.3389/fcvm.2026.1878487

**Published:** 2026-08-04

**Authors:** Laura Stanciulescu, Ancuta Nastac, Maria Dorobantu

**Affiliations:** 1 Department of Cardiothoracic Pathology, Carol Davila University of Medicine and Pharmacy, Bucharest, Romania; 2 Department of Medical Sciences, The Romanian Academy, Bucharest, Romania

**Keywords:** cardiovascular disease, catheter ablation, elderly, electrical storm, structural heart disease, ventricular tachycardia

## Abstract

**Background:**

Ventricular tachycardia (VT) catheter ablation is an established therapy in patients with structural heart disease (SHD), yet its use in elderly populations remains limited due to concerns regarding procedural risk and outcomes. This study aimed to evaluate the safety, efficacy, and clinical outcomes of VT ablation across different age groups.

**Methods:**

We performed a retrospective analysis of 142 consecutive patients with SHD who underwent radiofrequency catheter ablation (RFCA) for sustained monomorphic VT between 2013 and 2025. Patients were stratified into three age groups: ≤64 years (*n* = 71), 65–74 years (*n* = 40), and ≥75 years (*n* = 31). Primary endpoints included procedural safety and acute procedural success. Secondary endpoints assessed at 24 months were arrhythmic recurrence, appropriate implantable cardioverter-defibrillator (ICD) therapies, cardiovascular hospitalizations (CVH), and all-cause mortality.

**Results:**

Acute procedural success rates were similar across age groups (complete success: 76.05% vs. 72.5% vs. 77.42%, *p* = 0.87), with no significant differences in minor or major complication rates. At 24 months, arrhythmic recurrence (36.62% vs. 40% vs. 45.16%, *p* = 0.68) and appropriate ICD therapies (32.4% vs. 35% vs. 25.8%, *p* = 0.74) were comparable between groups. In contrast, CVH increased significantly with age (43.66% vs. 57.5% vs. 70.96%, *p* = 0.02), as did all-cause mortality (8.45% vs. 22.5% vs. 25.8%, *p* = 0.03).

**Conclusions:**

VT ablation in elderly patients with SHD demonstrates comparable procedural safety and arrhythmic efficacy to younger populations. However, advanced age is associated with worse long-term outcomes, likely reflecting a higher burden of comorbidities. These findings support an individualized approach to patient selection, where age alone should not preclude consideration for VT ablation in appropriately selected patients.

## 1 Introduction

Ventricular tachycardia (VT) represents a leading cause of mortality in patients with structural heart disease (SHD) (1). Implantable cardioverter-defibrillators (ICDs) are widely used in this population and effectively reduce sudden cardiac death (SCD) by terminating VT episodes through anti-tachycardia pacing (ATP) and shocks (2–4). However, ICDs do not prevent the occurrence of VT, and shocks—beyond being distressing for patients—have been linked to poorer survival (5, 6). As a result, strategies aimed at reducing VT burden and ICD therapies are essential, particularly in patients with recurrent arrhythmias (7–9). In this context, catheter ablation (CA) has become an established and effective approach to decrease VT episodes and appropriate ICD interventions (10–14). Although it is still debatable whether VT ablation improves long-term survival in general, it can be acutely life-saving in the setting of incessant VTs and electrical storm (ES) (1).

Advances in public health and medicine have contributed to increased life expectancy and a progressively aging population, bringing a distinct spectrum of diseases and clinical challenges (1). Although CA is an established and effective treatment for ventricular arrhythmias, it remains an invasive procedure and is often underutilized in elderly patients due to their higher baseline mortality risk and greater burden of comorbidities. Nevertheless, in patients with a reasonable life expectancy and clear indications for the procedure, most studies agree that age alone should not be a limiting factor in offering catheter ablation. But in clinical practice when it comes to VT ablation, is age truly just a number—or is there a threshold beyond which it becomes a limiting factor?

Studies on this topic have yielded inconsistent results. While some report similar arrhythmic recurrence and mortality rates between elderly and younger patients, others indicate that older individuals face higher risks of complications, rehospitalization, mortality, and longer hospital stays (1). These discrepancies may partly reflect heterogeneity in the definition of “elderly,” ranging from patients aged ≥65 years to those ≥80 years, as well as differences in patient selection. In particular, variability in comorbidity burden and frailty—common in older populations—may significantly influence procedural outcomes, quality of life, and overall prognosis.

Given the underrepresentation of older adults in large clinical trials and the growing relevance of frailty, there is a clear need to better define evidence-based management strategies for this expanding population. We therefore aimed to evaluate the feasibility and clinical outcomes of VT catheter ablation in a contemporary elderly cohort with SHD, in comparison with a younger population with similar baseline characteristics.

## 2 Methods

### 2.1 Study population

This retrospective single-center observational cohort study initially screened 159 consecutive patients who underwent VT ablation at the Cardiac Electrophysiology Department of a tertiary cardiovascular center between 2013 and 2025. Patients were excluded if they had idiopathic VT (*n* = 7), acute ischemia-related VT (*n* = 2), primary electrical disease (*n* = 5), or incomplete follow-up/missing records (*n* = 3). The final study cohort included 142 consecutive patients with SHD aged 12 to 96 years (mean 63.2 ± 14.3 years; 80.3% male), who underwent radiofrequency catheter ablation (RFCA) for sustained monomorphic ventricular tachycardia (SMVT).

Prior to ablation, all patients underwent a comprehensive evaluation, including echocardiography, stress testing, coronary angiography, computed tomography (CT), or late-gadolinium enhanced cardiac magnetic resonance (LGE-CMR), to identify the SHD phenotype, assess ventricular function, and delineate areas of interest.

**Figure 1:**
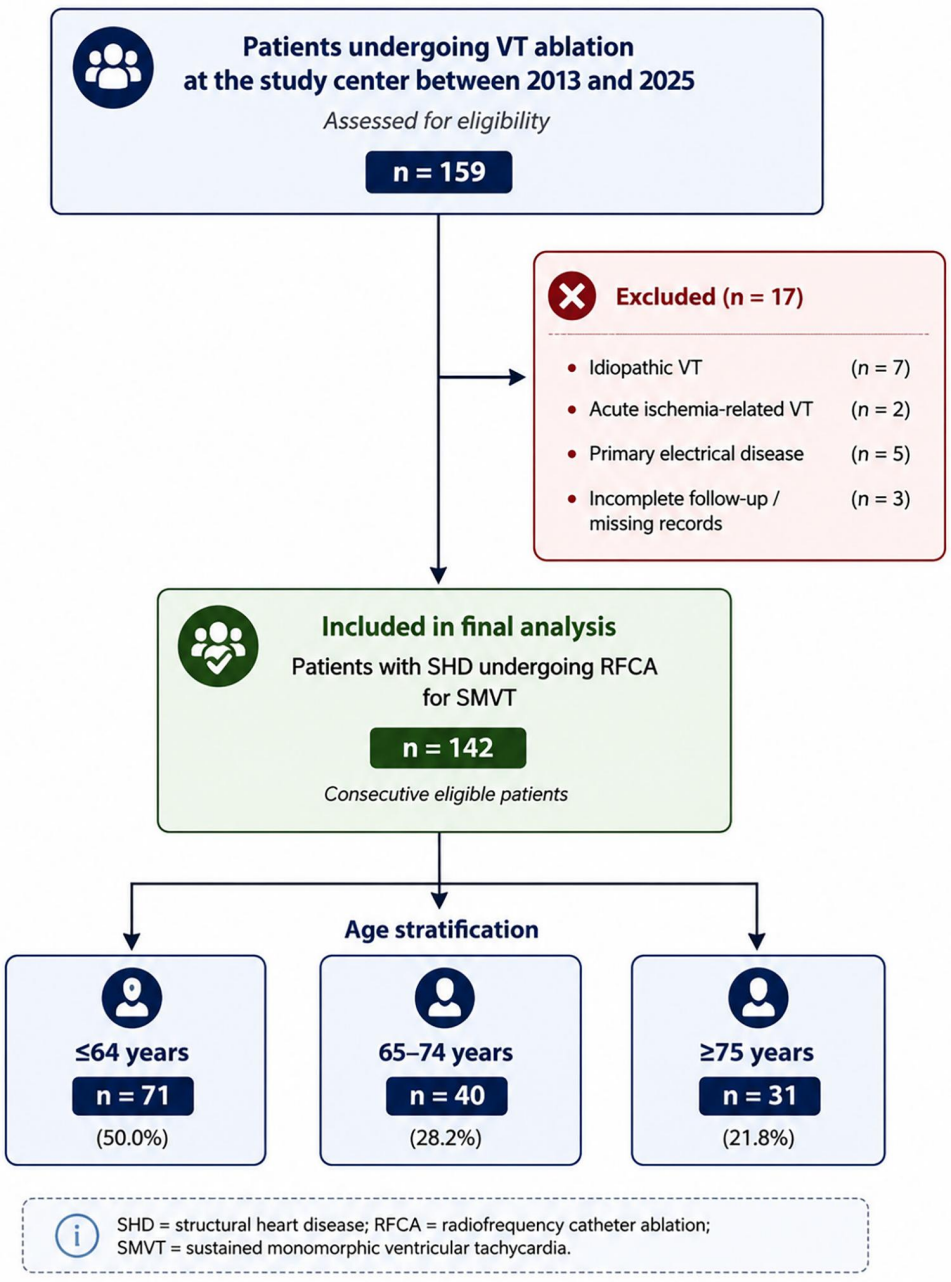
STROBE flow diagram illustrating patient screening, exclusions, and final cohort selection.

Classification into ischemic cardiomyopathy (ICM) or nonischemic cardiomyopathy (NICM) was primarily based on the presence of significant coronary artery disease (CAD) confirmed by coronary angiography. NICM was defined in accordance with the 2023 European Society of Cardiology Guidelines for the management of cardiomyopathies (3). The study cohort comprised 82 patients with ICM and 60 patients with NICM, while individuals without SHD were excluded from analysis. A detailed overview of patient screening, exclusions, and final cohort selection is presented in the STROBE flow diagram (Figure 1).

ES was defined as a condition of severe ventricular electrical instability, manifested by three or more sustained ventricular arrhythmias within 24 h, with each separated by at least five minutes and requiring therapeutic termination, according to the current guidelines (3). Chronic kidney disease (CKD) was defined as a glomerular filtration rate below 60 mL/min/1.73 m^2^.

To enable a comprehensive comparison across age groups, the study population was stratified into three categories: patients younger than 65 years, those aged 65–74 years, and those aged 75 years or older. All participants provided written informed consent for the procedure, following institutional protocols. The study was performed with the approval of the institution's ethics committee and in accordance with the declaration of Helsinki.

### 2.2 Electrophysiology study and VT ablation

All patients underwent electrophysiological study (EPS) and RFCA under conscious sedation after an overnight fast. Intracardiac signals were recorded and interpreted using the Boston Scientific Labsystem PRO EP platform (v.2.7.0.16). Electroanatomical substrate mapping was performed during sinus rhythm with the CARTO-3 system (Biosense Webster, Diamond Bar, CA, USA), using bipolar signal filtering between 16 and 500 Hz. High-resolution maps were systematically acquired, typically including more than 1,800 points, with focused point density in scarred myocardium and adjacent border-zone regions.

Catheter access was individualized according to the ventricular substrate and target chamber. Right ventricular (RV) mapping was performed through transfemoral venous access, whereas left ventricular (LV) access was obtained using either a transseptal or retrograde transaortic approach. In selected patients with suspected epicardial substrate involvement, epicardial mapping was achieved through fluoroscopy-guided anterior subxiphoid percutaneous puncture. Additional use of remote magnetic navigation (Niobe II, Stereotaxis Inc., St. Louis, MO, USA) or multielectrode mapping catheters, including decapolar and duodecapolar systems, was left to operator preference.

Myocardial substrate characterization relied on established voltage thresholds. Healthy endocardial tissue was defined by bipolar electrogram amplitudes >1.5 mV, with normal unipolar amplitudes defined as >8.3 mV in the left ventricle and >5.5 mV in the right ventricle. For epicardial mapping, normal bipolar voltage was considered >1 mV. Dense scar and border-zone tissue were identified by endocardial bipolar voltages <0.5 mV and between 0.5–1.5 mV, respectively. Scar dimensions, including dense scar burden and total scar extent, were quantified directly on the final electroanatomical maps using the CARTO-3 integrated measurement software, while border-zone proportions were subsequently derived from these values.

A predominantly substrate-oriented ablation strategy was adopted, aiming to eliminate conduction channels located within the scar border-zone. Radiofrequency applications were delivered using open- irrigated catheters with power settings ranging from 35 to 50 W and a target temperature up to 45 °C.

In cases where the VT was either spontaneously present or inducible and sufficiently tolerated from a hemodynamic standpoint, activation and entrainment mapping techniques were additionally used to define critical isthmus regions within the reentry circuit.

Following completion of substrate modification, programmed ventricular stimulation (PVS) was systematically performed to evaluate residual arrhythmia inducibility. Stimulation protocols incorporated at least two drive cycle lengths (CL) with up to four extrastimuli; in patients with advanced, volatile HF, stimulation was limited to three extrastimuli. Coupling intervals were reduced to a minimum of 200 ms or until ventricular refractoriness was reached. Stimulation was generally delivered from two separate sites adjacent to the scarred substrate. If SMVT with CL ≥250 ms remained inducible, additional substrate modification was performed after reassessment of residual conducting channels. Persistent inducibility of SMVT at the end of the procedure was considered indicative of residual arrhythmogenic substrate. Clinical VT was defined according to concordance with previously documented 12-lead ECG morphology or ICD intracardiac electrograms demonstrating comparable CL (±20 ms). For patients undergoing multiple procedures, procedural analyses were based on the final ablation intervention.

Complications were classified according to the 2019 HRS/EHRA/APHRS/LAHRS expert consensus on catheter ablation of ventricular arrhythmias (3). Major complications were defined as events leading to prolonged hospitalization or readmission, requiring additional therapeutic intervention, or resulting in serious injury or death. All other events, including minor hematomas not necessitating intervention, were considered minor complications.

### 2.3 Follow-up protocol

Follow-up information was collected based on each patient's most recent VT ablation procedure in cases where multiple interventions had been performed. Data were obtained through review of hospital records and routine ICD interrogations conducted every six months. For patients who were not followed at our center, clinical outcomes and ICD interrogation results were gathered from their referring cardiologists.

All-cause mortality was assessed retrospectively from the date of the most recent ablation, without distinction between cardiovascular and non-cardiovascular deaths. However, cardiovascular hospitalizations (CVHs) during the follow-up period were recorded separately from hospitalizations due to other causes. VT recurrence was defined as episodes of SMVT or ventricular fibrillation (VF) that were appropriately managed by the ICD, either through ATP or device-delivered shocks. ICD detection settings were programmed to identify any ventricular arrhythmias that had been previously observed or inducible during PVS (with a rate threshold 20 bpm below the slowest known VT), while slower arrhythmias outside these parameters were not actively monitored.

Missing data were limited and primarily affected follow-up variables derived from ICD interrogations in patients monitored outside the study center. Analyses for each endpoint were performed using available-case analysis, without data imputation.

### 2.4 Outcomes

Secondary outcomes were assessed during a follow-up (FU) period of 24 months after the index ablation procedure. These included arrhythmic recurrence, defined as the occurrence of sustained ventricular arrhythmias requiring device therapy, and the burden of appropriate ICD therapies, including both ATP and ICD shocks. Additional secondary endpoints comprised CVHs occurring during the FU period and all-cause mortality.

### 2.5 Statistical analysis

Continuous variables are reported as mean ± standard deviation or as median with interquartile range, according to their distribution. The normality of data was evaluated using the Kolmogorov–Smirnov test. Comparisons between groups were conducted using either the Student's t-test or the Mann–Whitney U test, as appropriate.

Categorical variables are presented as absolute counts and percentages and were compared using the chi-square test or Fisher's exact test. Effect sizes are expressed as mean differences (MD) for continuous variables and odds ratios (OR) for categorical variables, each accompanied by 95% confidence intervals.

Time-to-event outcomes were analyzed using Cox proportional hazards regression models to estimate age-stratified predicted event probabilities for arrhythmic recurrence, appropriate ICD therapies, and all-cause mortality during follow-up. Cox regression–derived event probability curves were subsequently constructed according to age group. Given the relatively limited sample size and event burden, the analyses were focused on outcome estimation across age categories in order to reduce the risk of model overfitting.

To minimize selection bias, all consecutive eligible patients during the study period were included. However, given the retrospective single-center design, residual confounding, referral bias, and selection bias related to patient eligibility for VT ablation cannot be completely excluded.

*P* values were calculated to assess differences across the three age groups using one-way analysis of variance (ANOVA) for continuous variables and the chi-square test for categorical variables; Fisher's exact test was applied when expected cell counts were low. All statistical tests were two-sided, with a *p* value <0.05 considered statistically significant. Statistical analyses were performed using SPSS version 25 (IBM Corp., Armonk, NY, USA).

## 3 Results

### 3.1 Patient characteristics

A total of 142 patients were included in the analysis and stratified into three age groups: ≤64 years (*n* = 71), 65–74 years (*n* = 40), and ≥75 years (*n* = 31). The overall cohort was predominantly male (80.3%), with a similar sex distribution across age groups. As expected, mean age differed significantly between groups. The prevalence of cardiovascular risk factors and comorbidities increased with advancing age, with significantly higher rates of hypertension (HTN), dyslipidemia, and atrial fibrillation (AF) observed in older patients. In contrast, the distribution of type 2 diabetes mellitus (T2DM), CKD, smoking status, and prior revascularization procedures was comparable across groups. ICM was more frequent in older individuals.

Regarding clinical status at admission, no significant differences were observed in NYHA functional class distribution, although a trend toward more advanced classes was noted in the oldest group.

Hemodynamic (HD) stability during VT episodes differed between groups, while the proportion of patients with an ICD and those receiving antiarrhythmic or beta-blocker therapy at the time of the procedure was similar. Left ventricular ejection fraction (LVEF) showed a modest but significant variation across age groups.

Detailed baseline characteristics are presented in Table 1.

**Table 1:** Baseline characteristics of the study population.

Baseline characteristic	All	Patients aged 64 years old or less	Patients aged 65–74 years old	Patients aged 75 years old or more	*p*-value
	(*n* = 142)	(*n* = 71)	(*n* = 40)	(*n* = 31)	
Males	80.28% (114)	77.46% (55)	82.5% (33)	83.87% (26)	0.71
Age	63 ± 14	52.2 ± 11.2	69.5 ± 3.3	80 ± 5	<0.001
Hypertension	68.31% (97)	54.93% (39)	80% (32)	83.87% (26)	0.004
T2DM	23.94% (34)	18.31% (13)	25% (10)	35.48% (11)	0.23
CKD	27.46% (39)	19.71% (14)	35% (14)	35.48% (11)	0.18
Dyslipidemia	73.24% (104)	59.15% (42)	87.5% (35)	87.1% (27)	0.002
History of AF	32.4% (46)	23.94% (17)	27.5% (11)	58.06% (18)	0.003
History of ES	72.53% (103)	73.24% (52)	80% (32)	61.3% (19)	0.18
Active smoker	23.24% (33)	19.71% (14)	30% (12)	22.58% (7)	0.47
ICM	57.74% (82)	46.47% (33)	75% (30)	61.3% (19)	0.02
Prior PCI	44.36% (63)	39.43% (28)	47.5% (19)	51.61% (16)	0.55
Prior CABG	5.63% (8)	2.81% (2)	5% (2)	12.9% (4)	0.11
Prior cardiac surgery (other than CABG)	2.11% (3)	1.4% (1)	5% (2)	0% (0)	0.29
Amiodarone at the time of the procedure	62.67% (89)	61.97% (44)	65% (26)	61.3% (19)	0.92
Beta-blocker at the time of the procedure	87.32% (124)	84.5% (60)	92.5% (37)	87.1% (27)	0.46
NYHA I at admission	4.93% (7)	9.86% (7)	0% (0)	0% (0)	0.03
NYHA II at admission	36.62% (52)	40.84% (29)	37.5% (15)	25.8% (8)	0.32
NYHA III at admission	45.77% (65)	45.25% (30)	48.05% (19)	51.61% (16)	0.84
NYHA IV at admission	12.67% (18)	7.04% (5)	15% (6)	22.58% (7)	0.12
Hemodynamically stable while in VT	88.73% (126)	95.77% (68)	77.5% (31)	87.1% (27)	0.01
ICD present	63.38% (90)	61.97% (44)	67.5% (27)	61.3% (19)	0.81
LVEF	34.2 ± 12.26	36.8 ± 13.3	29.7 ± 10.6	33.8 ± 10	0.01

### 3.2 Procedural details

Procedural and electrophysiological characteristics were broadly comparable across age groups. The number of clinical VT episodes, as well as the number of clinical and inducible VT morphologies, did not differ significantly between groups. Similarly, clinical VT CL, number of ablation procedures, and overall procedural duration were consistent across age categories. The distribution of ablation strategies (endocardial, epicardial, and combined endo-epicardial) was also similar, with a predominance of endocardial approaches in all groups. The extent of substrate involvement, assessed by the number of affected AHA segments, showed no significant variation. Notably, RV involvement differed significantly between groups, being more prevalent in younger patients. Acute procedural outcomes, including both partial and complete success rates, were comparable across all age groups.

Detailed procedural characteristics are summarized in Table 2.

**Table 2:** Procedural characteristics of the study population.

Procedural characteristic	All	Patients aged 64 years old or less	Patients aged 65–74 years old	Patients aged 75 years old or more	*p*-value
	(*n* = 142)	(*n* = 71)	(*n* = 40)	(*n* = 31)	
Number of clinical VT episodes	15.8 ± 23.91	14.52 ± 23.42	17.35 ± 24	16.76 ± 25.67	0.88
Number of clinical VT morphologies	1.17 ± 0.47	1.1 ± 0.34	1.27 ± 0.64	1.2 ± 0.47	0.36
Number of inducible VT morphologies	2.09 ± 1.77	1.94 ± 1.71	2.5 ± 2.2	1.9 ± 1.13	0.28
Clinical VT cycle length (ms)	373.76 ± 54.22	371.21 ± 58.7	370.24 ± 52.21	384.15 ± 45.73	0.41
Number of ablation procedures	1.39 ± 0.85	1.5 ± 1.02	1.35 ± 0.58	1.22 ± 0.66	0.32
Procedural duration (min)	223.68 ± 72.88	224.53 ± 63.7	232.15 ± 96.94	210.8 ± 54.9	0.53
Endocardial ablation	69.71% (99)	60.56% (43)	80% (32)	77.42% (24)	0.06
Epicardial ablation	2.11% (3)	2.81% (2)	0% (0)	3.22% (1)	0.57
Endo-epicardial ablation	28.16% (40)	36.62% (26)	20% (8)	19.35% (6)	0.08
Number of involved AHA segments	3.63 ± 1.77	3.72 ± 1.77	3.75 ± 2	3.27 ± 1.41	0.47
Presence of RV involvement	21.12% (30)	35.21% (25)	5% (2)	9.67% (3)	0.001
Overall acute procedural success	88.73% (126)	91.55% (65)	90% (36)	80.64% (25)	0.21
Acute complete procedural success	75.35% (107)	76.05% (54)	72.5% (29)	77.42% (24)	0.87
Acute partial procedural success	13.38% (19)	15.49% (11)	17.5% (7)	3.23% (1)	N/A
Acute procedural failure	11.27% (16)	8.45% (6)	10% (4)	19.35% (6)	N/A

### 3.3 Periprocedural adverse events

A total of 10 major complications were observed, including four cases requiring intraprocedural cardiopulmonary resuscitation (CPR) and two episodes of cardiogenic shock. Additional major events comprised one periprocedural myocardial infarction (MI) due to coronary artery injury during radiofrequency delivery, one transient ischemic attack (TIA) occurring during the procedure, one TIA within 7 days without an identifiable alternative cause, and one case of left bundle branch block (LBBB) progressing to HF.

Seventeen minor complications were documented, including two groin hematomas and 15 cases of pericarditis without clinical or echocardiographic signs of tamponade. Complications were more frequent in patients with NICM, accounting for half of the major events and 13 of the minor complications.

Two deaths were recorded in the NICM group: one occurred intraprocedurally due to refractory cardiogenic shock, and the other during the index hospitalization as a result of procedure-related sepsis secondary to aspiration pneumonia (Table 3).

**Table 3:** Summary of procedural complications.

Complication	All	Patients aged 64 years old or less	Patients aged 65–74 years old	Patients aged 75 years old or more	*p*-value
	(*n* = 142)	(*n* = 71)	(*n* = 40)	(*n* = 31)	
Minor complications	11.97% (17)	14.08% (10)	12.5% (5)	6.45% (2)	0.53
Groin hematoma	1.4% (2)	2.81% (2)	0% (0)	0% (0)	0.39
Pericarditis	10.56% (15)	11.26% (8)	12.5% (5)	6.45% (2)	0.76
Major complications	7.04% (10)	5.63% (4)	10% (4)	6.45% (2)	0.72
CPR during procedure	2.81% (4)	1.4% (1)	5% (2)	3.22% (1)	0.36
Cardiogenic shock	1.4% (2)	0% (0)	5% (2)	0% (0)	0.18
Periprocedural MI	0.7% (1)	1.4% (1)	0% (0)	0% (0)	0.56
TIA during the procedure	0.7% (1)	1.4% (1)	0% (0)	0% (0)	0.56
TIA <7 days after procedure	0.7% (1)	1.4% (1)	0% (0)	0% (0)	0.56
LBBB resulting in HF	0.7% (1)	0% (0)	0% (0)	3.22% (1)	0.56

### 3.4 Long-term outcomes

Follow-up data were available for the majority of patients; however, a small proportion of missing data was observed for certain outcomes, primarily due to incomplete ICD interrogations or follow-up performed outside the study center. Analyses were therefore conducted based on available data for each endpoint.

**Figure 2:**
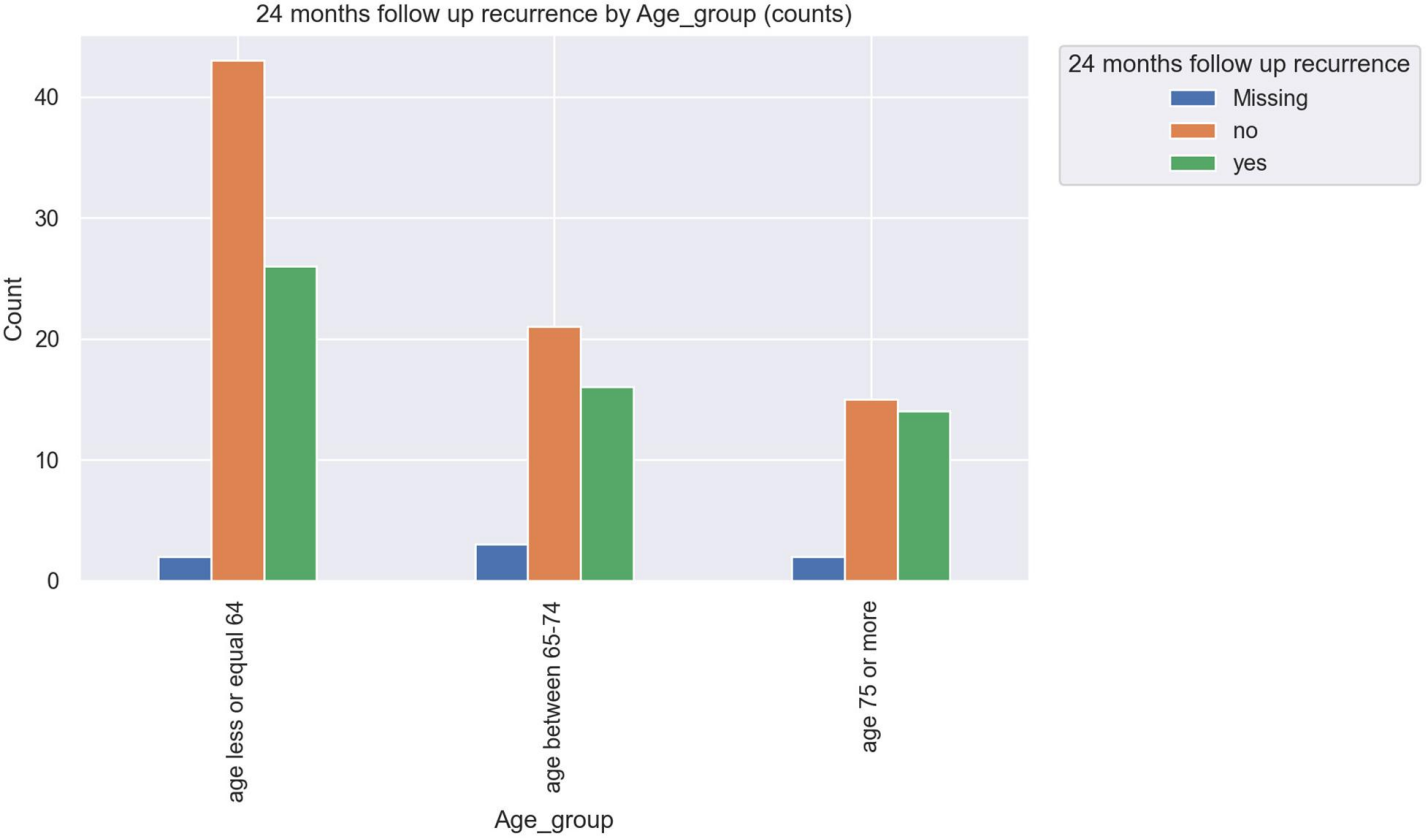
Twenty–four–month arrhythmic recurrence according to age group.

During a follow-up period of 24 months, arrhythmic recurrence was observed in 39.4% of patients, with no significant differences across age groups (Figure 2).

**Figure 3:**
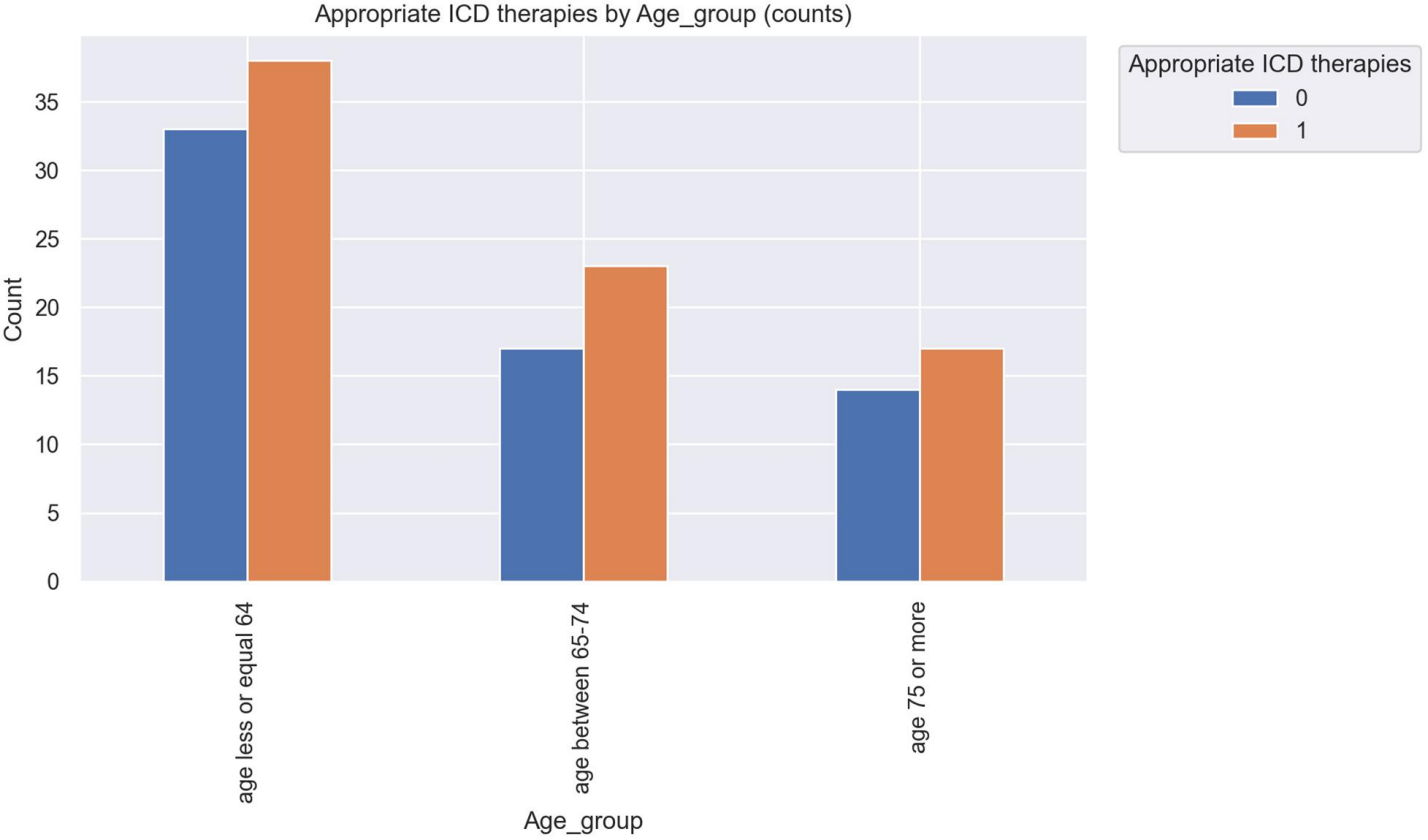
Incidence of appropriate ICD therapies at 24 months according to age group.

Similarly, the incidence of appropriate ICD therapies, including both ATP and device-delivered shocks, was comparable between groups, suggesting a similar post-ablation arrhythmic burden irrespective of age (Figure 3). Percentages for ICD-related outcomes are calculated based on available data.

**Figure 4:**
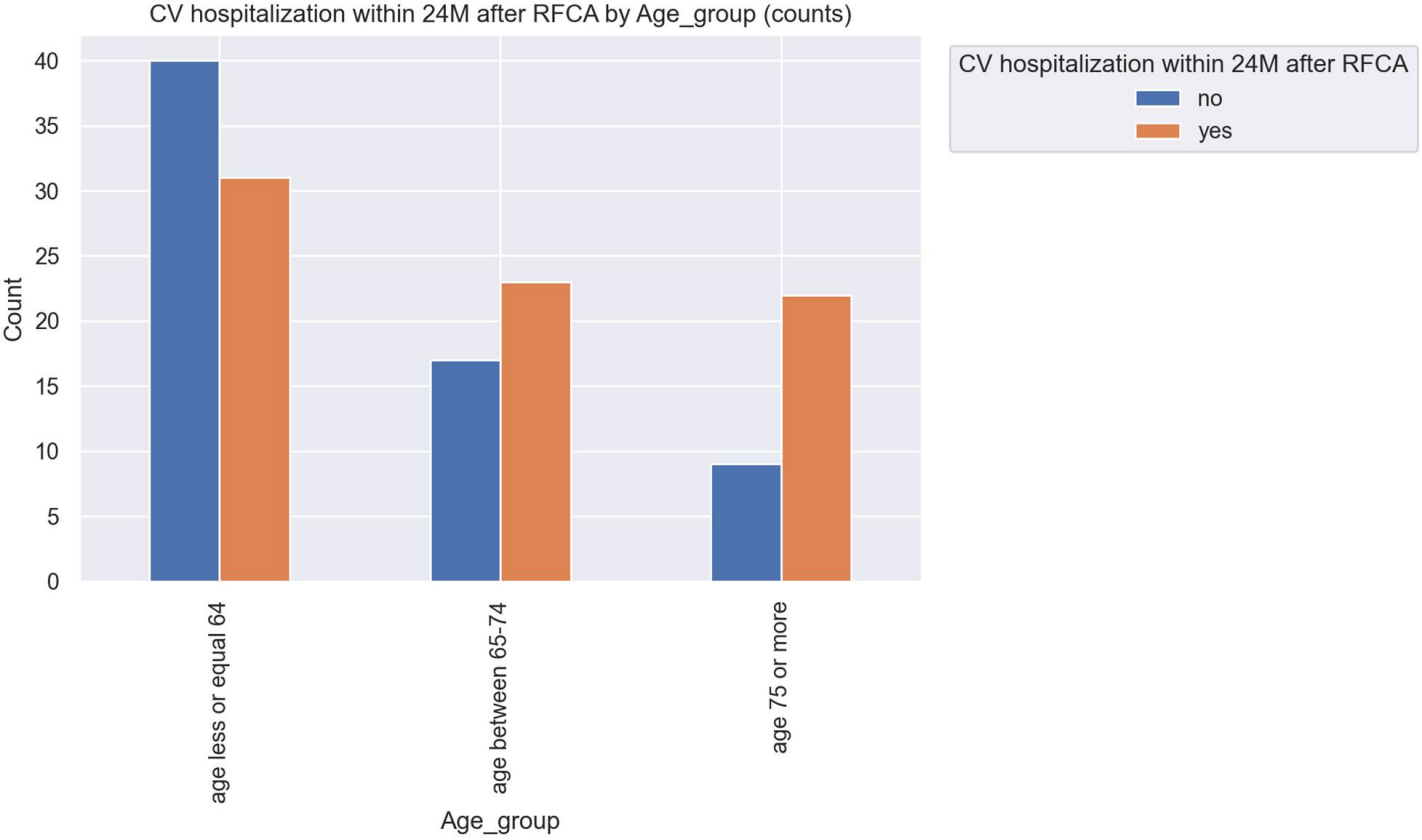
Cardiovascular hospitalizations within 24 months after radiofrequency catheter ablation for ventricular tachycardia according to age group.

**Figure 5:**
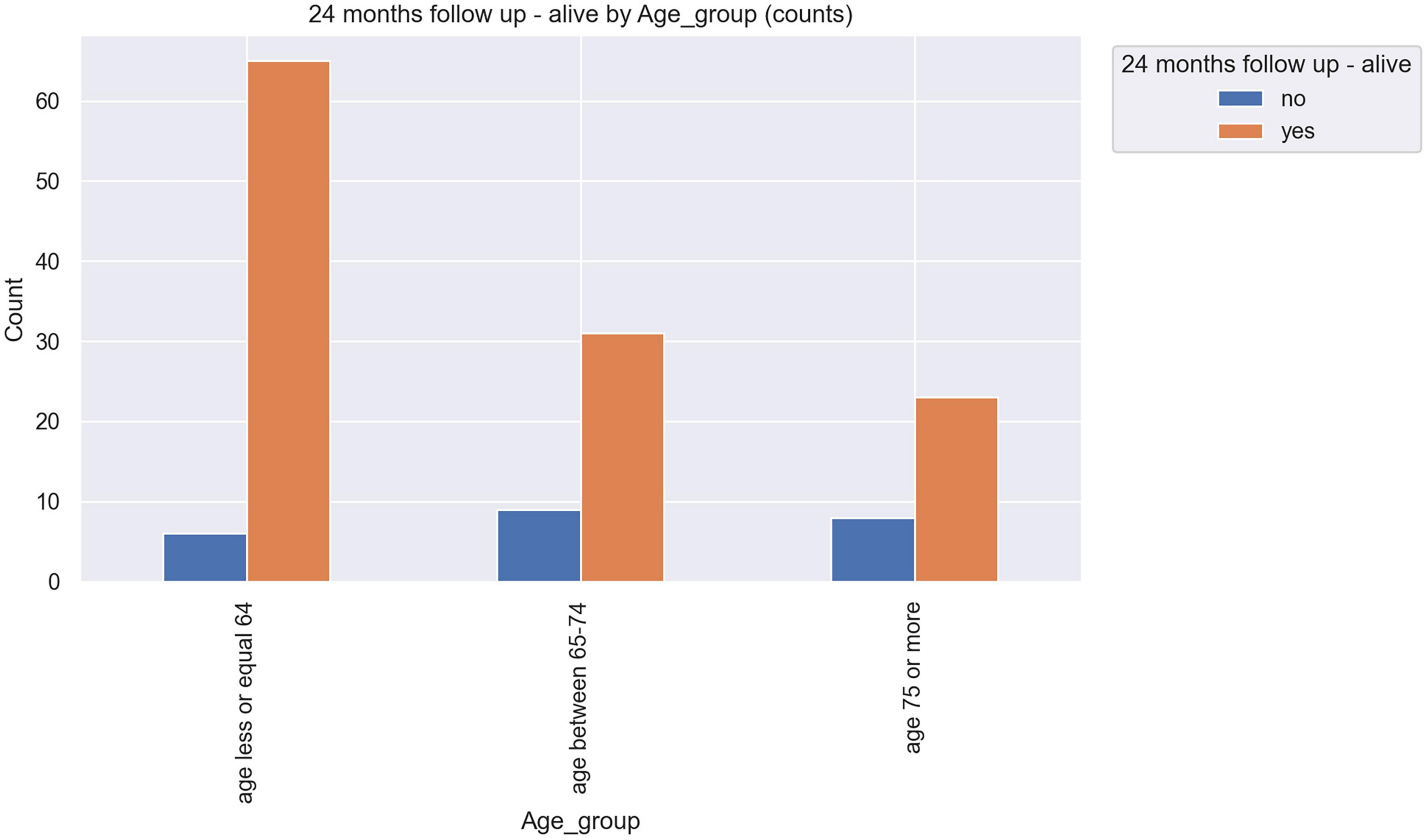
Twenty-four-month all-cause mortality according to age group.

In contrast, CVHs occurred more frequently with advancing age, reaching the highest proportion in patients aged ≥75 years, and this difference was statistically significant (Figure 4). A similar trend was observed for all-cause mortality, which was significantly higher in older patients compared to younger cohorts (Figure 5).

**Figure 6:**
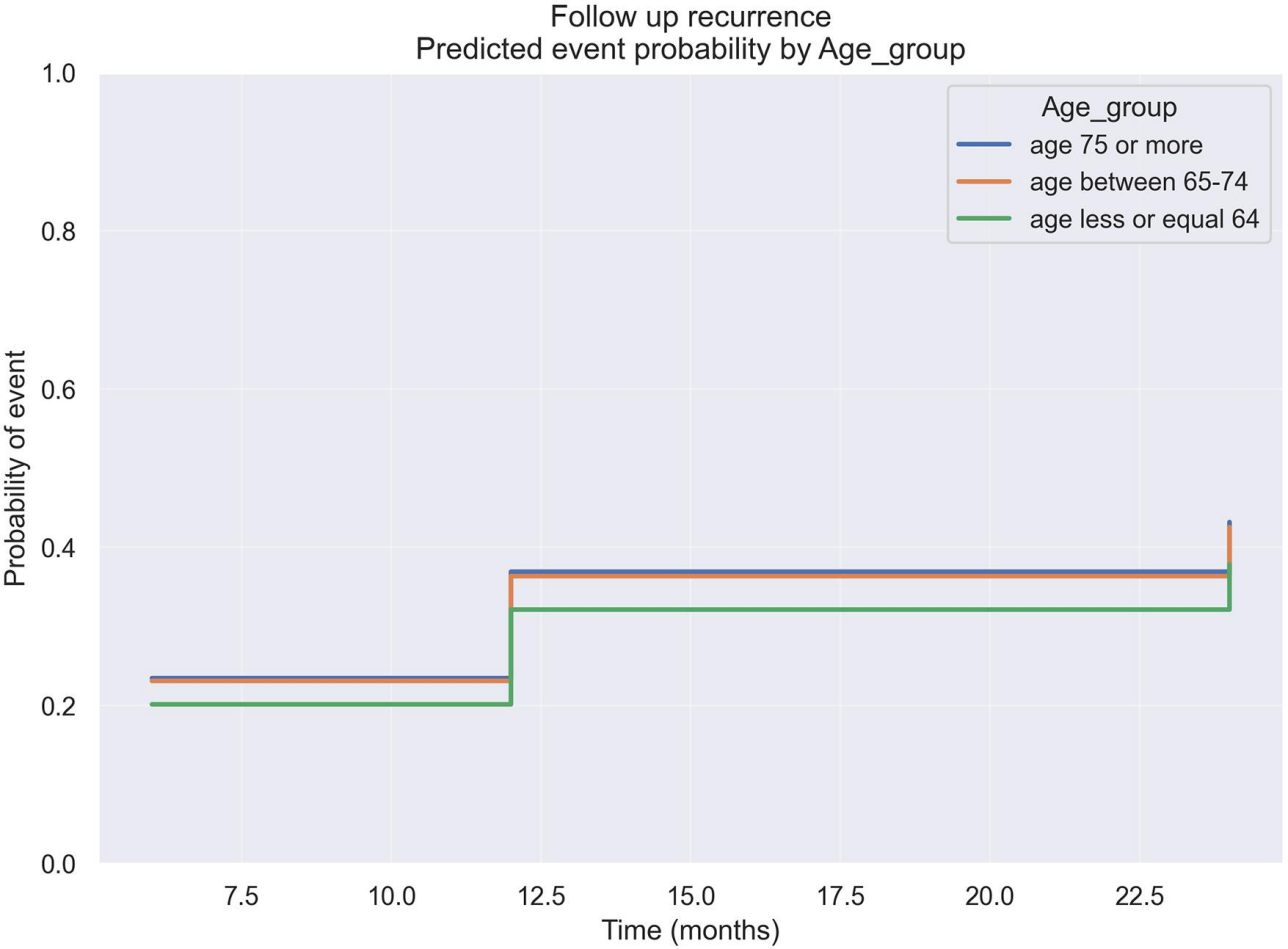
Cox regression-based predicted event probability curves for arrhythmic recurrence.

**Figure 7:**
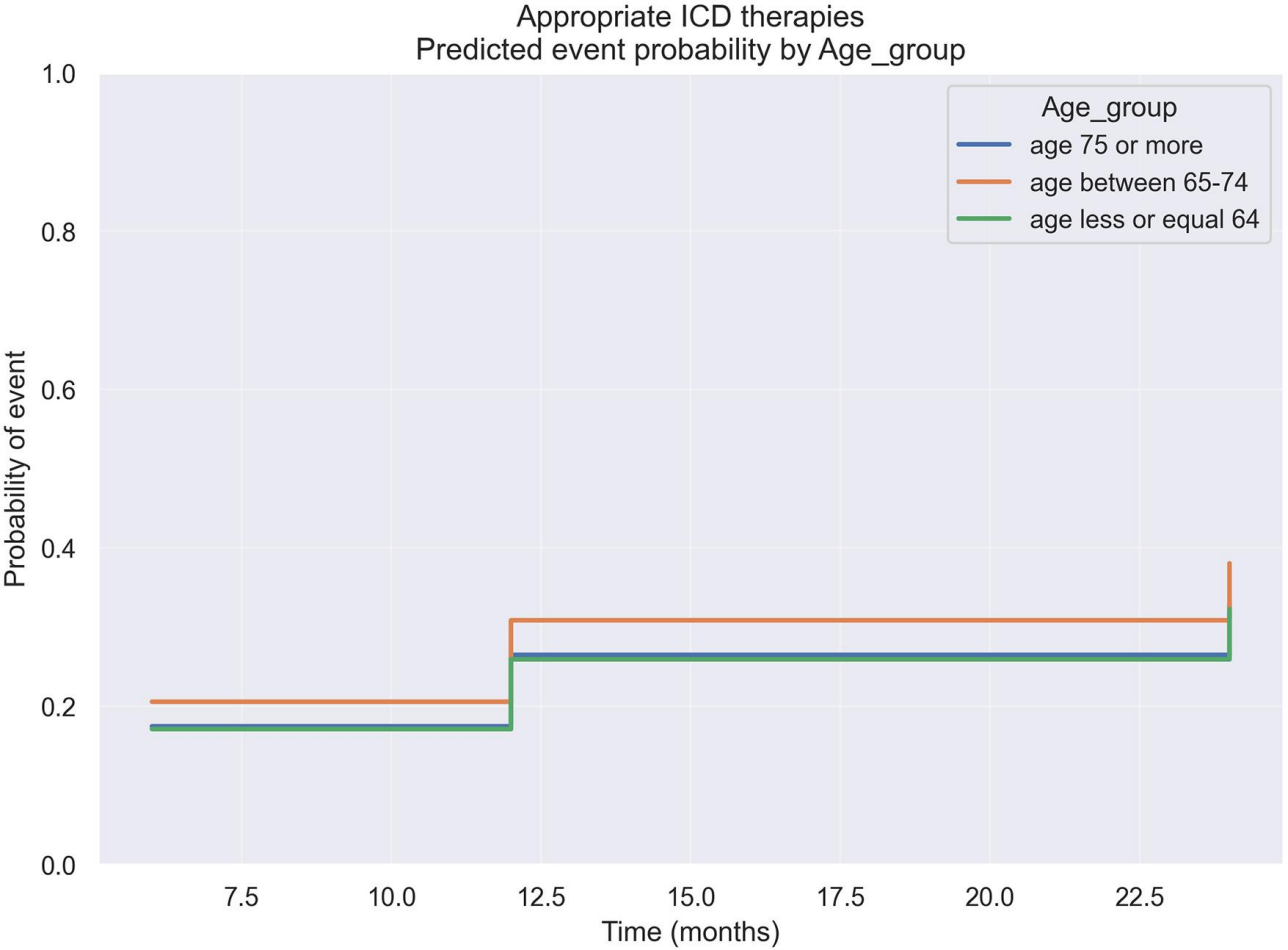
Cox regression-based predicted event probability curves for appropriate ICD therapies.

**Figure 8:**
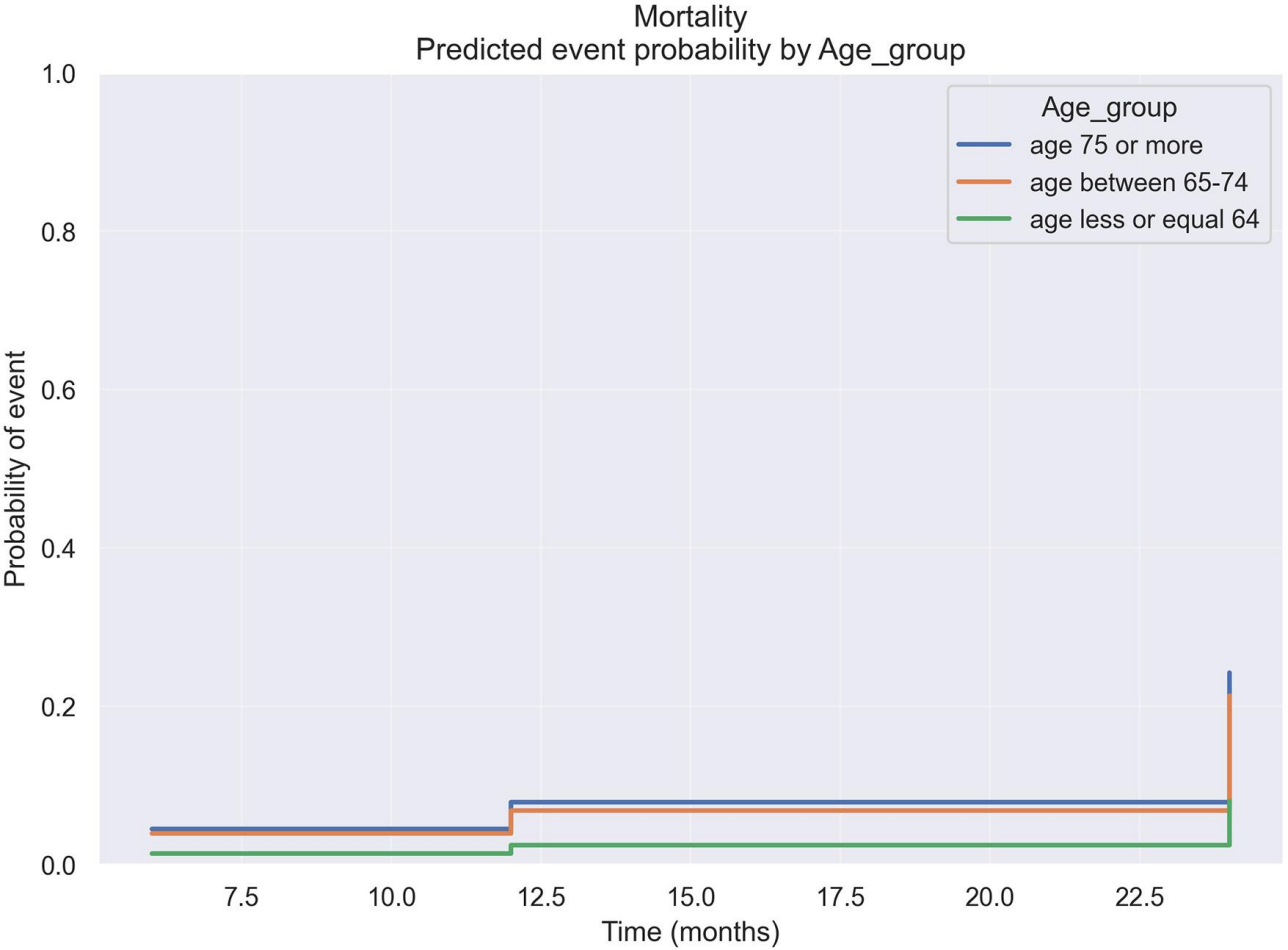
Cox regression-based predicted event probability curves for all-cause mortality.

To further assess the temporal distribution of long-term outcomes across age groups, we constructed Cox regression–based predicted event probability curves for arrhythmic recurrence, appropriate ICD therapies, and all-cause mortality. The predicted probability of arrhythmic recurrence remained largely comparable between age groups during follow-up, with only a modest tendency toward higher recurrence rates in older patients (Figure 6). Similarly, the probability of appropriate ICD therapies, including both ATP and ICD shocks, did not show meaningful differences according to age after VT ablation (Figure 7). In contrast, the mortality curves demonstrated a progressive increase in predicted risk with advancing age, with the highest estimated probability observed in patients aged ≥75 years (Figure 8).

Overall, while arrhythmic outcomes and ICD therapy burden were comparable across age groups, older patients exhibited a higher risk of adverse clinical events during follow-up, particularly in terms of CVH and mortality (Table 4).

**Table 4:** Long-term clinical outcomes at 24 months according to age group.

Clinical outcome	All	Patients aged 64 years old or less	Patients aged 65–74 years old	Patients aged 75 years old or more	*p*-value
	(*n* = 142)	(*n* = 71)	(*n* = 40)	(*n* = 31)	
Arrhythmic Recurrence	39.43% (56)	36.62% (26)	40% (16)	45.16% (14)	0.68
Appropriate ICD Therapies	31.7% (45)	32.4% (23)	35% (14)	25.8% (8)	0.74
Appropriate ATP	30.98% (44)	30.98% (22)	35% (14)	25.8% (8)	0.79
Appropriate ICD Shocks	23.24% (33)	23.94% (17)	22.5% (9)	22.58% (7)	0.99
Cardiovascular Hospitalization	53.52% (76)	43.66% (31)	57.5% (23)	70.96% (22)	0.02
All-cause Mortality	16.2% (23)	8.45% (6)	22.5% (9)	25.8% (8)	0.03

## 4 Discussion

### 4.1 Main findings

In this age-stratified cohort of patients with SHD undergoing RFCA for SMVT, the main finding is that increasing age was not associated with a clear reduction in acute procedural efficacy or with a significantly higher rate of periprocedural complications.

Acute partial and complete procedural success were comparable across the three age groups, and neither minor nor major complication rates differed significantly between younger patients, those aged 65–74 years, and those aged ≥75 years. Similarly, at 24 months, arrhythmic recurrence, and appropriate ICD therapies, including ATP and ICD shocks, were not significantly different according to age.

These findings suggest that, in appropriately selected patients, chronological age alone does not appear to compromise the immediate effectiveness or procedural safety of VT ablation. This is particularly relevant given the historical reluctance to refer elderly patients for invasive electrophysiological procedures, largely due to concerns regarding frailty, comorbidities, and perceived procedural risk.

**Figure 9:**
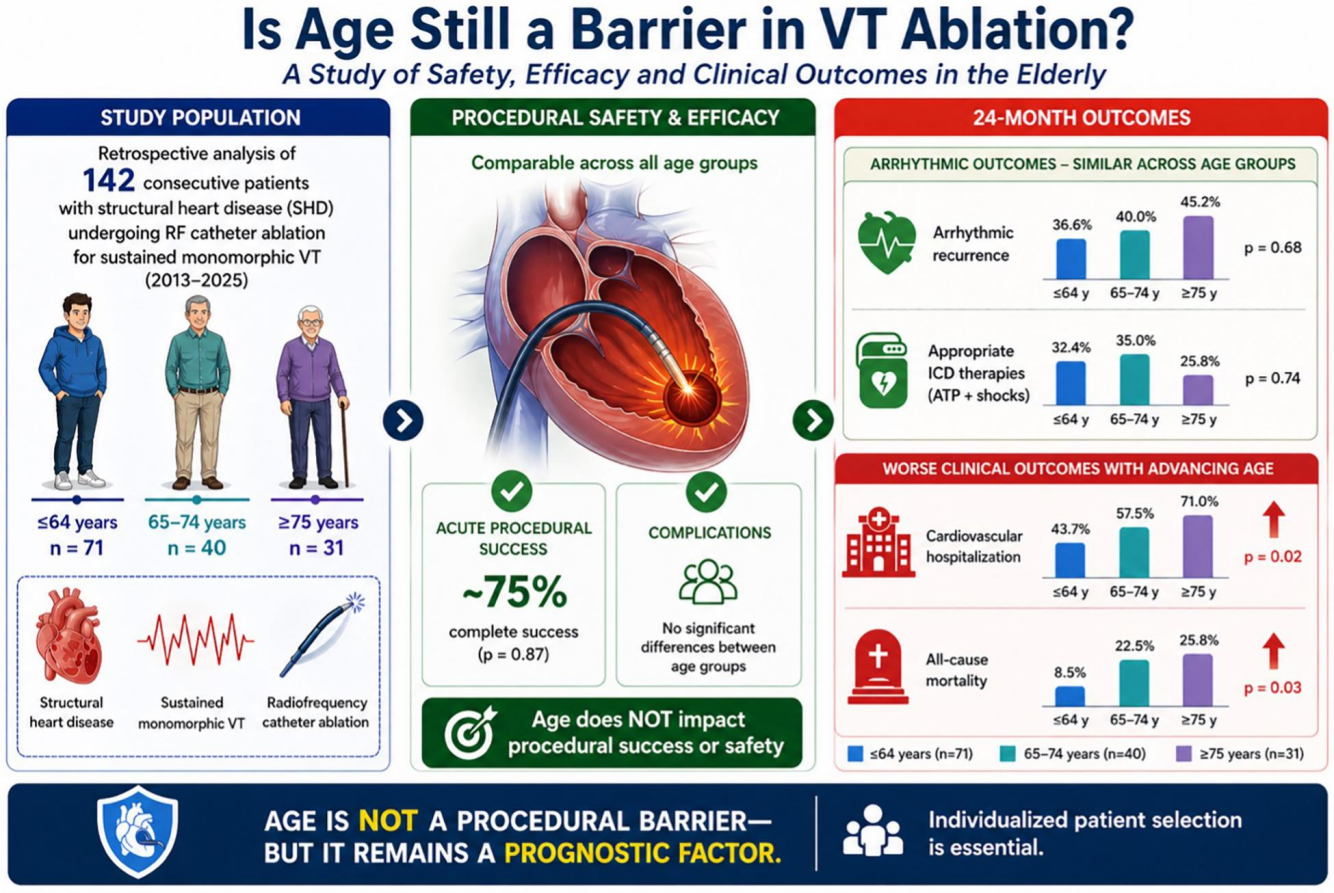
Graphical abstract of the study.

At the same time, older age was associated with less favorable long-term clinical outcomes. CVHs and all-cause mortality were significantly more frequent in older patients, particularly in those aged ≥75 years. Therefore, the present data support an important distinction: while age did not appear to reduce arrhythmic efficacy or procedural success, it remained associated with worse global prognosis during follow-up. This suggests that the excess risk observed in elderly patients may be driven less by ablation failure itself and more by the broader burden of cardiovascular disease, frailty, and competing non-arrhythmic risks. A graphical summary of the principal findings of the present study is provided in Figure 9.

### 4.2 Interpretation and comparison with literature

The role of VT ablation has evolved considerably over the past two decades. ICDs remain central to the prevention of SCD in patients with SHD, but they do not prevent VT occurrence and may expose patients to recurrent device therapies. ICD shocks, in particular, have been associated with worse survival and impaired quality of life, reinforcing the need for strategies that reduce VT burden and ICD interventions (1–6). Current guidelines and expert consensus documents recognize RFCA as an established treatment for recurrent SMVT, particularly in patients with SHD, recurrent ICD therapies, or ES (3, 15). Randomized trials and meta-analyses have further shown that VT ablation can reduce recurrent VT, ICD therapies, and VT storm, even though its effect on long-term mortality remains less certain (7–14, 16).

Within this framework, the management of elderly patients remains less clearly defined. Older adults are often underrepresented in randomized trials, and observational data have reported heterogeneous results. Some studies suggest that VT ablation in elderly patients achieves procedural success and recurrence outcomes comparable to those observed in younger cohorts, whereas others report higher rates of complications, rehospitalization, or mortality (17–29). This inconsistency is partly explained by differences in study design, patient selection, definitions of advanced age, cardiomyopathy substrate and procedural era.

The findings of the present study are broadly consistent with prior reports suggesting preserved procedural efficacy in elderly patients. Liang et al. reported that VT ablation in elderly patients with SHD was feasible, with acceptable safety and efficacy outcomes (21). Vakil et al., in an international multicenter analysis, also demonstrated that elderly patients undergoing VT ablation had meaningful arrhythmic benefit, although mortality remained influenced by age and underlying disease burden (22).

Similarly, studies focusing on octogenarians and very elderly populations have suggested that ablation can be performed with acceptable procedural risk in selected patients, even though advanced age remains associated with greater vulnerability and worse long-term outcomes (20, 23, 25, 28).

The present results also align with the systematic review and meta-analysis by Blandino et al., which found that elderly patients had similar acute ablation success and long-term VT recurrence compared with younger patients, but higher overall complications, periprocedural mortality, and long-term mortality (17). In contrast to that meta-analysis, our cohort did not demonstrate a significant age-related increase in procedural complications. This may reflect patient selection, procedural strategy, operator experience, or the use of contemporary mapping and ablation technologies. However, the higher rates of CVH and all-cause mortality observed in older patients are consistent with the broader literature and emphasize that procedural success does not fully offset the adverse prognostic impact of age-related comorbidity.

Our results also complement contemporary data on in-hospital outcomes after VT ablation. Tan et al. reported that age may influence hospital outcomes after VT ablation, highlighting the importance of careful risk assessment in older individuals (18). Inada et al. similarly showed that catheter ablation in elderly patients with coronary artery disease can be performed, but mortality remains substantial and strongly related to the severity of underlying disease (19). Taken together, these studies, as well as our own findings support a more nuanced interpretation: VT ablation should not be withheld solely because of age, but age should remain part of a broader clinical assessment that includes comorbidity burden, ventricular function, frailty, arrhythmic presentation, and expected benefit.

### 4.3 Mechanisms and rationale

Several mechanisms may explain why elderly patients in this cohort achieved similar procedural and arrhythmic outcomes despite worse long-term clinical prognosis. First, VT ablation directly targets arrhythmogenic substrate and re-entrant circuits responsible for recurrent SMVT. When the arrhythmic substrate is identifiable and accessible, particularly in scar-related VT, procedural success may be achievable regardless of chronological age. This may explain why acute procedural success and 24-month arrhythmic recurrence did not differ significantly across age groups.

Second, the use of modern EAM, substrate-based ablation, and systematic PVS may reduce some of the historical limitations associated with VT ablation in older patients. Contemporary approaches allow better characterization of scar architecture, identification of abnormal electrograms, and more targeted substrate modification, which may be particularly useful when VT is poorly tolerated or unmappable. These advances are reflected in current expert consensus recommendations and may help explain the comparable procedural outcomes observed in this cohort (11, 15).

Third, the worse long-term outcomes observed in older patients likely reflect factors beyond recurrent VT alone. In the present study, older groups had a higher prevalence of hypertension, dyslipidemia, AF, and ICM, while CVHs and all-cause mortality increased with age. This supports the concept that elderly patients may derive arrhythmic benefit from ablation while remaining vulnerable to HF progression, ischemic burden, frailty, and competing cardiovascular risks. Therefore, similar VT recurrence rates should not be interpreted as equivalent overall prognosis.

The underuse of VT ablation in elderly patients is also clinically relevant. Advanced age is often associated with therapeutic hesitation, especially when patients present with multiple comorbidities or reduced physiological reserve. However, the present findings support the view that age alone is an insufficient reason to deny referral for VT ablation in appropriately selected patients. Rather, decisions should be individualized and based on procedural indication, arrhythmia burden, substrate characteristics, patient goals, frailty, and anticipated quality-of-life benefit. This approach is consistent with the broader direction of contemporary cardiovascular medicine, in which chronological age is increasingly viewed as less informative than biological age and clinical vulnerability (30–34).

### 4.4 Strengths and limitations

The main strength of this study is its age-stratified analysis of a contemporary cohort of patients with SHD undergoing RFCA for SMVT. By separating patients into three clinically meaningful age categories, the study provides a more granular view of procedural safety, efficacy, and long-term outcomes than analyses based only on a single elderly vs. non-elderly cutoff. Our cohort also includes detailed baseline, electrophysiological, procedural, complication, and 24-month follow-up data, allowing assessment of both arrhythmic and broader clinical outcomes.

Furthermore, we believe there is an added value in our study's ability to distinguish between procedural efficacy and long-term prognosis. In our cohort, elderly patients did not experience significantly higher complication rates or reduced acute success, nor did they have higher rates of arrhythmic recurrence or appropriate ICD therapies at 24 months. However, they did experience more CVHs and higher all-cause mortality. This distinction is clinically important because it suggests that VT ablation may remain effective in elderly patients, while their overall prognosis continues to be shaped by comorbidities and competing cardiovascular risks, suggesting that, while age should inform clinical decision-making, it should not function as an automatic exclusion criterion.

The main limitation of this study is its retrospective design and the relatively small number of patients, particularly in the cohort aged ≥75 years. There is potential for selection bias, as VT ablation may have been preferentially offered to healthier elderly patients or to those considered more likely to tolerate the procedure. Accordingly, the older patients included in this study likely represent a relatively selected population considered suitable for intervention rather than the broader elderly population with VT. This may partly explain the comparable prevalence of certain comorbidities across age groups and should be considered when interpreting the generalizability of our findings.

Additionally, there is no universally accepted age threshold to define “elderly” or “geriatric” patients, and prior studies have applied varying cutoffs, making direct comparison challenging. The study cohort also includes procedures performed over an extended period, during which mapping systems, ablation strategies, operator experience, and peri-procedural care may have evolved. While this may have introduced some degree of temporal heterogeneity, further stratification by procedural era was not performed due to the limited sample size after age-group subdivision.

An additional important limitation relates to sample size within specific age and clinical subgroups. Although the overall cohort included 142 patients, the subgroup aged ≥75 years comprised only 31 individuals, with a relatively limited number of events for several outcomes. As a result, the statistical power to detect between-group differences was constrained, and robust multivariable adjustment was not feasible without substantial risk of model overfitting. Accordingly, the Cox regression-derived probability curves presented in this study should be interpreted conservatively, particularly in the oldest age group, as they are intended primarily for descriptive and exploratory visualization of temporal trends rather than definitive risk prediction.

Similarly, several clinically relevant subgroups were underrepresented, particularly female patients and patients with nonischemic cardiomyopathy. This limits subgroup-specific interpretation and may reduce the generalizability of our findings to these populations.

Another limitation is that this analysis was not designed to determine whether VT ablation provides a mortality advantage over antiarrhythmic therapy in patients aged ≥75 years. Such a question would require prospective randomized evaluation. All-cause mortality was assessed without systematic adjudication of cardiovascular and non-cardiovascular causes of death, limiting the ability to determine whether deaths were directly related to arrhythmic events, HF progression, procedural consequences, or competing non-cardiac conditions. Finally, some follow-up data, particularly ICD-related outcomes, were based on available device interrogations, and missing data may have influenced event estimates. ICD-related analyses were based on ICD status at the time of ablation, as post-procedural implantation data were not consistently available for all patients.

Additionally, this was a single-center study, and the findings may reflect center-specific referral patterns, procedural strategies, and operator experience, which may limit generalizability to other institutions and healthcare settings.

Overall, larger prospective multicenter studies incorporating standardized age definitions, frailty assessment, detailed cause-of-death adjudication, and contemporary ablation strategies are needed to validate our findings.

## 5 Conclusions

Our study demonstrates that VT ablation in elderly patients with SHD can be performed with procedural safety and arrhythmic efficacy comparable to that observed in younger individuals. However, advanced age remains associated with worse long-term clinical outcomes, particularly in terms of CVH and all-cause mortality, likely reflecting a higher burden of comorbidities and overall disease severity.

These findings support and individualized approach to patient selection, in which age is considered within the broader clinical context rather than serving as an independent limiting factor for VT ablation, especially in appropriately selected patients. Larger, multicenter, prospective trials are warranted in order to confirm these results.

## Data Availability

The raw data supporting the conclusions of this article will be made available by the authors, without undue reservation.
