# Supplementary material for: Is age still a barrier in ventricular tachycardia ablation? A study of safety, efficacy and clinical outcomes in the elderly
Source: Front Cardiovasc Med. 2026 Aug 4;13:1878487. doi: 10.3389/fcvm.2026.1878487 (PMC13481204; doi:10.3389/fcvm.2026.1878487)
Supplement: Supplementary file 1 [file Table1.docx]

**Supplementary Table 1. Cohort distribution by era**

| **Variable** | **2013–2019** | **2020–2025** |
| --- | --- | --- |
| Total patients (N) | 74 | 68 |
| Mean age ± SD | 63.1 ± 13.6 | 66.9 ± 17.3 |
| ≤64 years | 43.24% (32) | 57.35% (39) |
| 65–74 years | 39.19% (29) | 16.18% (11) |
| ≥75 years | 17.57% (13) | 26.47% (18) |
| Male sex | 83.78% (62) | 76.47% (52) |
| History of electrical storm | 60.81% (45) | 85.29% (58) |
| ICM | 81.08% (60) | 32.35% (22) |
| NICM | 18.92% (14) | 67.65% (46) |
| Mean LVEF ± SD | 34.6 ± 12.4 | 34.8 ± 12.6 |

**Supplementary Table 2. Primary efficacy endpoints distributed by era**

| **Variable** | **2013–2019** | **2020–2025** |
| --- | --- | --- |
| Complete success (n) | 74.32% (55) | 76.47% (52) |
| Partial success (n) | 13.51% (10) | 13.24% (9) |
| Failure / No success (n) | 12.16% (9) | 10.29% (7) |

**Supplementary Table 3. Primary safety endpoints distributed by era**

| **Variable** | **2013–2019** | **2020–2025** |
| --- | --- | --- |
| Major complications (n) | 10.81% (8) | 13.24% (9) |
| Minor complications (n) | 5.41% (4) | 8.82% (6) |
| No complications (n) | 83.78% (62) | 77.94% (53) |
